# Disorder and function: a review of the dehydrin protein family

**DOI:** 10.3389/fpls.2014.00576

**Published:** 2014-10-31

**Authors:** Steffen P. Graether, Kelly F. Boddington

**Affiliations:** Department of Molecular and Cellular Biology, University of GuelphGuelph, ON, Canada

**Keywords:** abiotic stress, cold, dehydration, dehydrins, intrinsically disordered proteins, late embryogenesis abundant, localization, structure

## Abstract

Dehydration proteins (dehydrins) are group 2 members of the late embryogenesis abundant (LEA) protein family. The protein architecture of dehydrins can be described by the presence of three types of conserved sequence motifs that have been named the K-, Y-, and S-segments. By definition, a dehydrin must contain at least one copy of the lysine-rich K-segment. Abiotic stresses such as drought, cold, and salinity cause the upregulation of dehydrin mRNA and protein levels. Despite the large body of genetic and protein evidence of the importance of these proteins in stress response, the *in vivo* protective mechanism is not fully known. *In vitro* experimental evidence from biochemical assays and localization experiments suggests multiple roles for dehydrins, including membrane protection, cryoprotection of enzymes, and protection from reactive oxygen species. Membrane binding by dehydrins is likely to be as a peripheral membrane protein, since the protein sequences are highly hydrophilic and contain many charged amino acids. Because of this, dehydrins in solution are intrinsically disordered proteins, that is, they have no well-defined secondary or tertiary structure. Despite their disorder, dehydrins have been shown to gain structure when bound to ligands such as membranes, and to possibly change their oligomeric state when bound to ions. We review what is currently known about dehydrin sequences and their structures, and examine the various ligands that have been shown to bind to this family of proteins.

## Abiotic stress response in plants

The inability of higher plants to move away from danger was likely a major force in the development of their stress responses. An area of considerable research interest is a plant's ability to respond to various abiotic stresses such as drought, high salinity, and cold. All three of these result in dehydration, that is, a reduction of the amount of free water available to the cell. One family of proteins that is expressed during dehydration stress has been named dehydration proteins (dehydrins). In this review, we will focus on what is currently known about the sequence make-up and structure of dehydrins in higher plants, and what has been demonstrated *in vitro* with regards to their potential *in vivo* protective functions. We begin with a brief overview of their discovery and examine the localization of dehydrins inside the cell. Dehydrins found in mosses (Mundy and Chua, 1988; Saavedra et al., 2006; Ruibal et al., 2012), algae (Labhilili et al., 1995; Li et al., 1998) and cyanobacteria (Close and Lammers, 1993; Kim et al., 2012) will not be described here.

There has been considerable interest in understanding the mechanism by which plants can survive dehydration stress in order to protect crops from damage and to increase the amount of arable land. A study using cotton seeds identified a whole family of proteins upregulated during dehydration stress, which the authors named Late Embryogenesis Abundant (LEA) proteins (Galau et al., 1986). As their name suggests, these proteins are highly abundant during the later stages of seed development, which gives the seeds the ability to tolerate drought. Subsequent studies have shown that LEA proteins are present in many plants and different plant tissues. LEA proteins consist of different conserved sequence motifs. The number of LEA groups depends on the classification system used, but is often defined as 6 or 7 groups (Tunnacliffe and Wise, 2007; Battaglia et al., 2008). LEAs have a high number of Ala, Gly, and Ser residues, and very few hydrophobic residues. Their highly hydrophilic nature results in the proteins lacking significant secondary structure. For more details on the LEA protein family, see reviews by Wise and Tunnacliffe (2004), Tunnacliffe and Wise (2007), Battaglia et al. (2008), Hincha and Thalhammer (2012). Our review will focus on dehydrins, which are classified as members of the LEA protein family D-11 or group 2 (Close, 1997).

The expression of dehydrins has long been correlated with several abiotic stressors including drought, salinity and cold. Additionally, osmotic and cold stress can be simulated by treatment with abscisic acid (ABA) (Talanova and Titov, 1994), where this association has been studied on both the mRNA and the protein level. Nylander et al. (2001) characterized a set of five dehydrins from Arabidopsis using Western blotting, and found that three were upregulated in response to cold and one was upregulated only by ABA treatment. The fifth dehydrin was expressed constitutively, but upregulated by ABA, cold and salinity (Nylander et al., 2001). Danyluk et al. (1998) showed that both wcor410 mRNA transcripts and protein levels were upregulated in response to cold. Although dehydrins are not all upregulated by the same stresses, there are many examples of dehydrin mRNA levels increasing due to various abiotic stressors (e.g., Cellier et al., 1998; Zhu et al., 2000).

The exact role of dehydrins in the plant has yet to be determined, but several physiological effects have been correlated with the presence of dehydrins. Electrolyte leakage across membranes due to cold stress is a common assay used to determine cold sensitivity; a lower level of leakage has been observed several times with high levels of dehydrin expression (Ismail et al., 1997; Hara et al., 2003; Xing et al., 2011). Hara et al. (2003) also investigated the effects of dehydrins on lipid peroxidation, and found that the citrus dehydrin CuCOR19 prevented the oxidation of liposomes most likely by scavenging reactive oxygen species. A very different role has been shown by Xie et al. (2012), where the MtCAS31 dehydrin interacted with the ICE1 transcription factor to reduce stomatal density. Overall, there are still many avenues that need to be explored before the exact role, or more likely, roles, of dehydrins can be determined.

## Sequence and architecture of dehydrins

Close et al. suggested the term “dehydrin” in 1989 (Close et al., 1989), although the first published dehydrin sequence was described in the previous year by Mundy and Chua (1988). Dehydrin sequences are highly modular, consisting of a variable number of conserved motifs interspersed with regions that are weakly conserved. As such, dehydrins vary considerably in molecular weight, with the smallest dehydrin being 9.6 kDa (Labhilili et al., 1995) and the largest being 70 kDa (Kim et al., 2012). Often the top end of dehydrin molecular weights is cited as 200 kDa; this value reflects the apparent molecular weight of the protein as shown by sodium dodecyl polyacrylamide electrophoresis (SDS-PAGE), rather than its chemical molecular weight. This discrepancy is due to dehydrins running anomalously large on SDS-PAGE because of their disordered structure (Receveur-Bréchot et al., 2005).

### Dehydrin sequences

Dehydrin sequences are usually described in terms of three commonly conserved motifs (Close, 1996). Strictly speaking, the dehydrin family is defined by the presence of a Lys-rich sequence motif. This segment, also known as the K-segment, has the sequence EKKGIMDKIKEKLPG. However, an inspection of a range of other reported dehydrin sequences shows that its conservation is not absolute. A comparison of all K-segment motif definitions in the literature suggests that no position is absolutely conserved (Veltri and Graether, unpublished results). The most conserved residues are in the core of this segment (Lys-Ile-Lys-Glu), with the Ile sometimes substituted with another non-aromatic, hydrophobic amino acid, and the Glu residue occasionally substituted with Asp, which is also negatively charged. The residues flanking this core are slightly more variable, with Glu-Lys-Lys often present at the N-terminal end of this motif, though sometimes the initial Glu is substituted with Gln. The C-terminal end is generally Leu-Pro-Gly, with the Gly sometimes substituted with Leu and Pro sometimes substituted with His. Another conserved motif has been termed the Y-segment due to the presence of a Tyr residue. The Y-segment consists of the sequence motif (V/T)D(E/Q)YGNP, with the Asp and Gly-Asn-Pro residues being highly conserved. The last common motif found in dehydrins is the S-segment, whose name reflects that it consists of 5–7 Ser residues in a row, and is often preceded by Ser-Asp.

In addition to these conserved motifs, dehydrins have the ϕ-segment. To a large degree, the definition of this segment is a catchall definition, since neither the sequences nor the lengths of the ϕ-segments are conserved. Broadly speaking, ϕ-segments are defined as all of the residues located between the conserved Y-, S-, and K-segments. An analysis of the amino composition of the Pfam00257 family of the ϕ-segments (i.e., all sequences without the Y-, S-, or K-segments) reveals that the top most common amino acids are Gly, Gln, and Thr (Table 1), while Phe, Cys, and Trp are present ≤1% of the time.

**Table 1:** **Φ-segment amino acid composition**.

**Amino Acid**	**% of all** Φ**-segment residues**
Ala	6.3
Arg	2.9
Asn	1.9
Cys	0.5
Gln	6.4
Glu	11.0
Gly	17.8
His	7.3
Ile	1.7
Leu	2.6
Lys	8.5
Met	1.6
Phe	1.1
Pro	4.0
Ser	5.0
Thr	10.0
Trp	<0.1
Tyr	2.7
Val	3.9

Several other motifs have been suggested for dehydrins, but their ubiquity in the dehydrin family has not yet been established. One motif that has been seen in several dehydrins is a Lys-rich segment, also known as the charged peptide (ChP) segment (Hara et al., 2005; Mouillon et al., 2006; Hundertmark and Hincha, 2008). This motif appears to consist of 1 or 2 segments of 3–5 Lys residues in a row, and is often preceded by negatively charged amino acids (Glu or Asp). This motif is of particular note since it has some similarity to the Lys-rich K-segments. Proposed roles for the ChP segment include nuclear targeting, acting as a phosphorylation target (for those ChP segments that include a Ser residue) (Ildikó, 2013), DNA binding (Hara et al., 2005), and chaperone activity (Mouillon et al., 2006), although none have been experimentally proven.

Hara et al. (2005) and Eriksson et al. (2011) both identified His-rich segments as having possible functional significance for dehydrins, though for different reasons. As detailed below in the ligand binding section, it was discovered that the His-rich motif HKGEHHSGDHH is able to bind metals (Hara et al., 2005). In contrast, another work found that many dehydrins have His–His or a His flanking the K-segments, which has an effect on membrane binding by dehydrins (Eriksson et al., 2011).

The arrangement of the Y-, S-, and K-segments can vary, but dehydrin architecture generally falls under one of five possibilities: K_n_, SK_n_, K_n_S, Y_n_SK_n_, and Y_n_K_n_ (Close, 1996). The range of n depends on the particular segment. The Y-segment, when present, is generally found as one copy, although two copies have been reported (Wisniewski et al., 1999). For the S-segment, only one copy of the serine-rich segment is found in dehydrins. The K-segment, by definition, must be present in at least one copy (Close, 1996), though in many dehydrin sequences it occurs more frequently. In one dehydrin (CAP85) it is present 11 times (Neven et al., 1993).

### Dehydrin architecture, localization, and abiotic stress

Given the various ways in which the conserved segments can come together, it is interesting to see whether the presence and absence of particular motifs may correlate with where the dehydrin localizes in the cell, and what particular abiotic stress triggers its expression. A summary of these data are shown in Table 2, where the dehydrins are listed in an order based on their YSK architecture. Dehydrins can be found in many locations in the cell, with the most likely places being the cytoplasm and the nucleus. Other locations include mitochondria, chloroplasts, and near the plasma membrane. Two SK_n_ dehydrins (Danyluk et al., 1998; Yang et al., 2014) were found near the plasma membrane, while a K_n_S dehydrin was found with the mitochondrial fraction (Hara et al., 2003). No other dehydrin architecture was seen at a membrane, implying that the Y-segment does not have a role in membrane protection. For the remaining architectures, no definitive localization pattern emerges. Phosphorylation of the S-segment can relocate a dehydrin from the cytosol to the nucleus (Goday et al., 1994). However, a K_n_ dehydrin (Houde et al., 1995), and two Y_n_K_n_ dehydrins (Wisniewski et al., 1999; Lin et al., 2012) were found in the cytoplasm and the nuclei. It is possible that an unidentified sequence element is responsible for these differences in localization.

**Table 2:** **Dehydrin architecture, localization, and abiotic stress regulator**.

**Protein Name**	**YSK Arch**.	**Localization**	**Abiotic stress**	**References**
PV-dhn	K_5_	Cyt	n/d	Yakubov et al., 2005
LTI30	K_6_	n/d	Cold	Nylander et al., 2001
WCS120	K_5_	Nuc/Cyt	Cold	Houde et al., 1995
DHN24	SK_3_	Nuc/Cyt	Cold	Szabala et al., 2014
SpDHN1	SK_3_	Cyt/Mem	Desic	Yang et al., 2014
CpDHN	SK_2_	n/d	Cold	Porat et al., 2004
Peudhn1	SK_2_	n/d	Cold	Caruso et al., 2002
COR47	SK_3_	n/d	Cold	Nylander et al., 2001
ERD14	SK_2_	n/d	Cold, Salt	Nylander et al., 2001
LTI29	SK_3_	n/d	Cold, Salt	Nylander et al., 2001
PgDHN1	SK_4_	n/d	Cold, Desic	Richard et al., 2000
WCOR410	SK_3_	Mem	Cold, Desic	Danyluk et al., 1998
CuCor19	K_3_S	Mito	Cold	Hara et al., 2003
AmDHN1	YSK_2_	Nuc/Cyt	Desic, Salt	Mehta et al., 2009
VrDHN1a	YSK_2_	n/d	Cold, Desic	Xiao and Nassuth, 2006
TAS14	YSK_2_	Nuc/Cyt	Salt	Godoy et al., 1994
Rab17	YSK_2_	Nuc/Cyt	n/d	Goday et al., 1994
Rab21	YSK_2_	Cyt	Desic	Mundy and Chua, 1988
VrDHN1	Y_2_K	Nuc/Cyt	Desic, Salt	Lin et al., 2012
PCA60	Y_2_K_9_	Nuc/Cyt	Cold	Wisniewski et al., 1999
52 and 63 kDa dehydrins	n/d	Mito	Cold	Borovskii et al., 2005
31 kDa dehydrin	n/d	Chloro	Const	Mueller et al., 2003
24 kDa dehydrin	n/d	Nuc/Cyt	Cold	Karlson et al., 2003
24 kDa dehydrin	n/d	Nuc/Cyt	Cold	Rinne et al., 1999

*Abbreviations: Cold, cold induced; Chloro, chloroplast; Const, constitutively induced; Cyt, cytoplasm; Desic, desiccation induced; Mito, mitochondrion; n/d, not determined; Nuc, nucleus; Salt, salt induced*.

The relationship between the YSK architecture and abiotic stress is more definitive. It is likely that the stress response pathway (signaling and transcription control) is a possible factor in determining dehydrin expression patterns in response to the various abiotic stresses (Wang et al., 2003). For the proteins that have been studied, the K_n_, SK_n_, and K_n_S proteins are largely upregulated by cold stress, although some are also upregulated by desiccation and salt (Table 2). For the Y_n_SK_n_ dehydrins, desiccation and salt are the dominant stresses which cause their increased production. For the Y_n_K_n_ dehydrins, one is upregulated by desiccation and salt (Lin et al., 2012), while the other is upregulated by cold (Wisniewski et al., 1999). While not an absolute pattern, the data in Table 2 suggest that the presence of the Y-segment is more important for protection from desiccation and salt stress but not cold stress. This concept also fits with the idea that the Y-segment is not involved with membrane binding (see above), since the most significant amount of damage from cold stress comes from damage to the membrane (Steponkus, 1984). Therefore, the Y-segment may have evolved to protect the cell from damage that is caused by desiccation and salt rather than by cold.

The purpose of investigating the relationship between YSK architecture and dehydrin localization is to provide potential guidance on each segment's role in protecting the plant. Despite numerous studies, mostly with individual dehydrins, the patterns are not entirely clear (Eriksson and Harryson, 2011). One possible reason for this is that the YSK-naming system does not take into account the minor motifs that have been observed or the variable ϕ-regions that may have some other, yet to be determined, role in localization. Complicating this is that the comparisons among dehydrins are from different plants, which may have different protective needs depending on the plant's structure. Two comprehensive, recent studies on dehydrin localization in *Vitis vinifera* (Yang et al., 2012) and in *Triticum aestivum* (Wang et al., 2014) compare dehydrins with different YSK architectures in one species using one stress protocol. In the *Vitis* study, four dehydrin genes were identified and the mRNA levels in tissue during stress were examined (Yang et al., 2012). Based on the YSK-nomenclature, these dehydrins are DHN1 (Y_2_SK_2_), DHN2 (SK_2_), DHN3 (SK_3_), and DHN4 (Y_3_SK_2_). During seed development (embryogenesis), which prepares them for drought stress, all four dehydrins were detected. Cold and heat stress upregulated DHN1 and DHN2, whereas only DHN1 was upregulated due to drought stress. In the *T. aestivum* dehydrin study (Wang et al., 2014), the authors also examined dehydrin expression in seedling leaves and roots during dehydration, cold, and salt stress. In this work, YSK_2_ transcription responded to dehydration but not low temperatures, whereas K_n_ dehydrins responded only to cold.

## Structure and flexibility of dehydrins

An inspection of the dehydrin sequence reveals a lack of hydrophobic residues. For a typical, globular protein, it is the hydrophobic core that drives protein folding; in the case of dehydrins, the presence of mostly polar and charged amino acids prevents the protein from forming a stable structure. Proteins that are natively unstructured, that is, they lack defined secondary and tertiary structure, are known as intrinsically disordered proteins (IDPs). IDPs and proteins with intrinsically disordered regions (IDRs) have been identified in many organisms (Tompa, 2002; Uversky, 2002a,b). This type of disorder is especially common in cell signaling proteins and transcription factors (Uversky, 2002a), but is also found in stress response proteins such as the LEA proteins (Wise and Tunnacliffe, 2004; Tunnacliffe and Wise, 2007; Battaglia et al., 2008; Hincha and Thalhammer, 2012). One advantage of dehydrins being disordered is their inability to denature. Globular protein denaturation involves the exposure of hydrophobic residues to the aqueous environment, where they may interact with the exposed hydrophobic residues on other proteins and begin to aggregate. In the case of dehydrins, their intrinsic disorder prevents them from denaturing during desiccation or at freezing temperatures, since they have no significant structure to lose and very few hydrophobic residues that could cause aggregation. Practically speaking, this lack of an ability to denature has been exploited in the purification of dehydrins by using boiling as a step to lyse cells and remove contaminating proteins (Livernois et al., 2009).

Data that dehydrins are IDPs come from several studies. Early experimental evidence included the inability of dehydrins to be crystallized (Lisse et al., 1996), a common property of disordered proteins. It was also observed that dehydrins migrate anomalously on polyacrylamide gels; the large hydrodynamic radius of these proteins causes the protein movement to be retarded in the gel compared to globular proteins (Receveur-Bréchot et al., 2005). Direct evidence for disorder in dehydrins has been obtained largely from circular dichroism (CD) studies.

A comparison of CD data from several dehydrins consistently shows a similar pattern, where there is a signal minimum located near 200 nm and a considerably weaker, second minimum near 222 nm. The signal near 200 nm represents random coil (i.e., the lack of regular secondary structure); the signal at 222 nm represents α-helicity, though some caution must be made with regard to this interpretation. This observation is often cited as showing that the K-segments form α-helices. Although this signal indicates that there is some helicity in dehydrins, it should not necessarily be interpreted as suggesting that a part of the dehydrin is helical all of the time. Instead, one must think of the protein possibly having transient helical structure or weak, imperfect helical structure.

This issue has been directly examined using nuclear magnetic resonance (NMR), a technique that can provide residue specific information on structure and residual structure in disordered proteins (Forman-Kay and Mittag, 2013). Chemical shifts were measured for a simple dehydrin construct consisting of the architecture K-ϕ-K (based on the *Vitis riparia* dehydrin sequence VrDHN1) (Xiao and Nassuth, 2006; Findlater and Graether, 2009). Chemical shifts are exquisitely sensitive measures of a protein's structure and environment. Using the secondary structure propensity (SSP) analysis program (Marsh et al., 2006), it was shown that the K-segments do not form stable helices (Hughes and Graether, 2011). Assuming that the SSP output represents percent structure (α-helix or β-sheet), the central residues of the K-segments are helical <5% of the time, with residues flanking the middle being helical <2% of the time. A similar analysis was performed with ERD14 dehydrin from *Arabidopsis thaliana* (Szalainé Ágoston et al., 2011) using secondary chemical shifts (Wishart et al., 1995). They reported that the K-segments showed 7–23% α-helicity. The higher calculated α-helical content for ERD14 compared to K_2_ may reflect the interpretation of secondary chemical shift analysis, which does not take partial secondary structure into account like SSP does (Marsh et al., 2006), and may therefore overestimate secondary structure content.

The NMR relaxation properties of K_2_ (Hughes and Graether, 2011) and ERD14 (Szalainé Ágoston et al., 2011) have also been examined. These types of NMR experiments examine the dynamics (flexibility) of proteins on a residue specific basis. For K_2_, the entire protein is highly flexible, as is expected for an intrinsically disordered protein. A comparison of the K-segments with ϕ-segment in K_2_ shows that the ϕ-segment is even more flexible than the K-segment. Heteronuclear NOE relaxation experiments of ERD14 also found that the K-segments were slightly more rigid than the ϕ-segments. Given their lack of conservation, it is unlikely that ϕ-segments have a direct functional role. We propose that this very high flexibility in the ϕ-region could allow the K-segments to optimally orient with their target ligands (Hughes and Graether, 2011), possibly to keep the K-segments apart, or give the protein a large hydrodynamic radius (Hughes et al., 2013).

## Dehydrin ligands

While IDPs such as dehydrins are highly disordered *in vitro*, they often gain structure when bound to a target, suggesting that some disordered proteins may be structured *in vivo* in the presence of their cognate ligands. One method of identifying potential ligands is to determine where the dehydrin is localizing within the cell. Using immunohistochemistry and *in situ* hybridization with antidehydrin antibodies, dehydrins were found in the cytoplasm (Asghar et al., 1994; Goday et al., 1994; Egerton-Warburton et al., 1997; Puhakainen et al., 2004), nucleus (Goday et al., 1994), and near the plasma membrane (Egerton-Warburton et al., 1997; Danyluk et al., 1998; Puhakainen et al., 2004). Using immunoelectron microscopy and subcellular fractionation techniques, Danyluk et al. (1998) also found that the wheat WCOR410 dehydrin is associated with the membrane as a peripheral membrane protein.

### Membrane binding by dehydrins

The results suggested that dehydrins are able to interact with membranes. The binding of dehydrins to membranes *in vitro* has been performed using a number of different membrane systems, including membrane-mimicking detergent micelles (Ismail et al., 1999; Hara et al., 2001; Koag et al., 2003, 2009; Soulages et al., 2003). In most of these studies, membrane binding was assessed by following the gain of structure by the dehydrins, suggesting that the intrinsically disordered dehydrins gain structure once bound to a membrane surface.

The first dehydrin study used the 26.5 kDa cowpea protein and examined changes in structure by using CD in the presence of SDS micelles (Ismail et al., 1999). The spectra showed that the large negative peak near 200 nm from the dehydrin decreases in negative intensity in the presence of micelles, while at the same time the weak negative minimum at ~220 nm becomes more negative. The loss of signal at ~200 nm represents the loss of coil structure and likely the loss of disorder, while the broad negative band centered around 222 nm represents the gain of helical structure. A follow-up study by the same research group with *Zea mays* dehydrin 1 (ZmDHN1) showed a similar gain in α-helicity in the presence of sodium dodecyl micelles (Koag et al., 2003). The ability to bind membranes was further examined in this work using lipid vesicles (also known as liposomes) consisting of lipids that had different headgroups with different charges. Lipid binding was assessed by monitoring the elution of the dehydrin from a gel filtration column in the presence and absence of liposomes (Koag et al., 2003). The early elution of the protein from the column corresponds to protein bound to the lipid, since liposomes are much larger than the dehydrins. The results showed that dehydrins bound to negatively charged lipids such as phosphatidyl serine (PS), phosphatidyl glycerol (PG), and phosphatidic acid (PA), but did not bind to phosphatidyl choline (PC). Among these lipids, ZmDHN1 interacted most strongly with the PA and PC:PA containing vesicles, where all of the protein was in the bound fraction (Koag et al., 2003). For PC:PG and PC:PS liposomes, dehydrin was found both in the bound and unbound elution fractions. Several reports have shown that dehydrins gain α-helical structure in the presence of negatively charged liposomes and micelles (Ismail et al., 1999; Hara et al., 2001; Koag et al., 2003; Soulages et al., 2003), however, several different results have suggested that the gain in helicity and the lack of binding to neutral lipids may not be a property of all dehydrins, such that many different modes of binding between these proteins and membranes may occur.

In the work by Kovacs et al. (2008), a 1:1 (mol:mol) ratio of PC:PS lipids was used to create liposomes. Using a mini-gel filtration column and buffer containing only 50 mM Tris (i.e., no NaCl), they demonstrated that *Arabidopsis* dehydrins ERD10 and ERD14 were able to interact with these liposomes (Kovacs et al., 2008), and that the addition of 800 mM NaCl reduced binding dramatically. Binding inhibition by salt suggests that these dehydrins are binding to the membrane through electrostatic interactions. However, examination of the CD spectra of both proteins shows that there is no gain of α-helicity, suggesting a different mode of binding compared to ZmDHN1 (Koag et al., 2003). Using Fourier-transformed infrared spectroscopy (FTIR), Rahman et al. (2013) showed that for *Thellungiella salsuginea* dehydrin 1 (TsDHN-1) the type and amount of structural change was dependent on lipid type, where they detected the gain of some β-strand structure for this protein when bound to lipid compositions mimicking the plasma membrane, mitochondrial membrane and chloroplast membrane (Rahman et al., 2010, 2013). It is challenging to reconcile the gain of β-strand structure compared to the α-helical structure seen in other studies for dehydrins bound to membranes, since the plasma membrane mimicking vesicles contain phospholipids. The chloroplast mimicking membranes are considerably different, since they primarily consist of the galactolipids monogalactosyldiacylglycerol (MGDG) and digalactosyldiacylglycerol (DGDG), which are neutral.

Several papers have reported that dehydrins are unable to bind to membranes containing only zwitterionic lipids consisting of only PC (Koag et al., 2003, 2009; Soulages et al., 2003) or phosphatidyl ethanolamine (PE) (Koag et al., 2003). This was further corroborated by the lack of change in structure in the presence of PC liposomes as measured by CD (Koag et al., 2003). The same lack of structural change was observed for Glycine max dehydrin 1 (GmDHN1) with liposomes consisting of dimyristoyl phosphatidylcholine (DMPC) (Soulages et al., 2003).

Although dehydrins are able to bind strongly to negatively charged membranes, the interaction of this protein with lipids is not always purely dictated by the presence of a negative charge. The study by Soulages et al. (2003) showed that no structural change occurred with *Glycine max* dehydrin 1 (GmDHN1) in the presence of DMPC liposomes and dimyristoyl phosphatidyl glycerol (DMPG) liposomes, despite the negative charge on the DMPG headgroups. GmDHN1 has only one 13-residue K-segment, and may therefore bind liposomes more weakly than the other studied dehydrins, which have two or more K-segments (Koag et al., 2003; Rahman et al., 2010; Eriksson et al., 2011). Likewise, using surface plasmon resonances, it was shown that LTI30 was able to bind to dioleoyl phosphatidyl glycerol (DOPG), and to dioleoyl phosphatidyl serine (DOPS), both of which are negatively charged, but also to dioleoyl phosphatidyl (DOPC), which is neutral (Eriksson et al., 2011). The DOPC interaction was reported to be weaker than the interaction with the other two lipid types. Any detected interaction with PC is surprising given the other studies that have not seen binding to lipids containing choline headgroups or gain of α-helical structure (Koag et al., 2003, 2009; Soulages et al., 2003). It is not clear if this represents a fundamental difference in the membrane binding mechanism of LTI30 compared to other dehydrins, or whether it represents surface plasmon resonance sensitivity in detecting a binding interaction (in the millimolar range; Hall et al., 1996). It is possible that LTI30 may be interacting with DOPC not through the positively charged K-segments, since the K-peptide did not bind to DOPC vesicles, whereas intact LTI30 did (Eriksson et al., 2011).

One particularly interesting lipid type that has been shown to not bind to dehydrins is phosphatidyl inositol (PI). The work by Koag et al. (2003) showed that PI, despite being a negatively charged lipid, does not bind this dehydrin. These lipids do have a carbohydrate headgroup, which may sterically prevent the dehydrin from reaching the negatively charged phosphate backbone. ZmDHN1 is able to bind to vesicles containing PI:PC lipids, perhaps because the presence of PC reduces the density of carbohydrates at the surface that would otherwise prevent binding. But this proposal does not apply to all dehydrins, since TsDHN1 is able to bind to liposomes containing only galactolipids (Rahman et al., 2010).

Some of these different, and apparently inconsistent, results may be partially explained by the different types of membranes used for a particular type of lipid. Examples include the use of liposomes of different sizes [100 nm large unilamellar vesicles (LUVs), small unilamellar vesicles (SUVs), LUVs with a range of sizes, detergent micelles], and different lamellemarity (unilamellar vesicles vs. multilamellar vesicles), all of which have the potential to alter liposome properties. A differential effect of liposome sizes on dehydrin binding to membranes has already been demonstrated. The work by Koag et al. (2003) examined the issue of membrane planarity and binding by dehydrins. Using ZmDHN1, the authors found that this dehydrin bound small, unilamellar vesicles more strongly than large unilamellar vesicles (LUVs). The authors suggest that this may be due to an increased curvature causing more defects in packing, which could allow dehydrins to bind more strongly to the liposome surface (Koag et al., 2003).

The binding of dehydrins to negatively charged lipid headgroups suggests that the positively charged K-segments are involved in the interaction. To determine their role in binding, Koag et al. (2009) constructed several derivatives of ZmDHN1 in which the first (ΔK_1_) or second (ΔK_2_) K-segments had been deleted, and one construct in which both K-segments had been deleted (ΔK_3_). Interaction of these protein constructs was assessed both directly in a binding assay to LUVs (PC:PA, 1:1 mol ratio), and indirectly through examining changes in secondary structure by CD when bound to PC:PA SUV, and to SDS micelles (Koag et al., 2009). The authors found that the ΔK_1_ and ΔK_2_ proteins both bound to vesicles and showed a gain in α-helicity. ΔK_1_ showed a smaller gain in helicity than ΔK_2_, suggesting that it could be more weakly bound to the vesicle, whereas the ΔK_3_ construct showed almost no structural change. In addition, the K-peptide alone gained α-helicity in the presence of SDS micelles and in the presence of PC:PA liposomes. The authors summed the CD spectra of ΔK_1_ and ΔK_2_ in the presence of liposomes together, and found that the resulting spectrum is approximately the same as intact ZmDHN1. Taken together, these experiments show that it is the K-segment that is responsible for binding to membranes (Koag et al., 2009).

Another study examined the role that residues flanking the K-segment may have in modulating the binding of dehydrins to the membrane (Eriksson et al., 2011). Specifically, they proposed that the His residues located on either side of the K-segments in LTI30 (a K_6_ dehydrin from *A. thaliana*) help to modulate membrane binding. The role of the His residues in the interaction was shown by changing the pH and by the use of K-segment peptides with and without two flanking His residues. Eriksson et al. (2011) showed that the addition of LTI30 induced vesicle aggregation, and that this aggregation was pH dependent. Above pH 6.5, the aggregation process lessened, suggesting that the His residues, which have a pKa typically at 6.5, are responsible for this process. However, His-His dipeptides do not flank all dehydrin K-segments, and the K-peptide alone has been shown to bind vesicles (Koag et al., 2009), indicating that these residues are not critical for membrane binding for other dehydrins.

Plant membranes have been shown to be vulnerable to desiccation, salt, and cold stresses, stresses which have been shown to upregulate dehydrin expression. This suggests that dehydrins could prevent this damage. The biochemical studies described here have shown that dehydrins bound most strongly to PA, which is only a minor plant lipid (1–2%). PC, the most abundant lipid in the plant plasma membrane, for the most part does not appear to bind dehydrins. There are, however, several reasons why it may be advantageous for membranes containing PA to be protected. As pointed out by Koag et al. (2003), PA levels in plants increase during drought stress (Moreau et al., 1998; Frank et al., 2000; Munnik, 2001) and salt stresses (Munnik et al., 2000). During stress, PA rich lipid rafts may form, therefore leaving the membranes more vulnerable to damage. As well, vesicles involved in membrane trafficking are rich in PA and PS (Liscovitch, 1995). Therefore, these potential lipid rafts in the plasma membrane and membrane-trafficking vesicles could be important targets for protection by dehydrins during abiotic stress (Koag et al., 2003).

The mechanism by which dehydrins stabilize membranes requires further discovery. In the case of *Arabidopsis* ERD10 and ERD14 (Kovacs et al., 2008), diphenyl hexatriene (DPH) was used to probe the effect of dehydrins on membrane fluidity. For these two dehydrins, no effect was discovered (Kovacs et al., 2008). This may not be surprising, since DPH partitions into the acyl chain region of the membrane. The hydrophilic and electrostatic nature of the K-segments likely causes them to bind the lipid headgroups, which would not necessarily change the fluidity in the inner part of the membrane. In the study by Eriksson et al. (2011), the authors also examined the effect of dehydrin binding on the phase transition temperature (T_m_) of the lipids. The transition temperature was measured using differential scanning calorimetry (DSC) with DMPC:DMPS (3:1 mol:mol) liposomes. As a negative control, Cor47 dehydrin was used, which does not bind to membranes and had no effect on membrane T_m_ (Eriksson et al., 2011). In contrast, the addition of LTI30 lowered the T_m_. These findings suggest that in the presence of dehydrins, the plant membranes would be able to maintain a fluid, and hence more functional, membrane phase at a lower temperature.

### Binding of other ligands to dehydrins

Several research groups have also shown that, in addition to membranes, different dehydrins are able to bind to many small ligands and ions. A study by Tompa et al. quantified the amount of water associated with a dehydrin using wide-line NMR relaxation (Tompa et al., 2006). The ERD10 and ERD14 dehydrins bound considerably more water than the bovine serum albumin control. It is possible that this is a special property of dehydrins or that it represents the very large surface area of a disordered protein that exposes polar side chains, allowing them to associate with water. The authors argue that the technique shows that dehydrins can bind a large amount of water and are also able to bind a large amount of solute ions. They state that this enables dehydrins to retain water and to buffer the increase in ionic concentration during desiccation stress (Tompa et al., 2006).

In addition to binding to water, dehydrins have been proposed to bind to ice and act as antifreeze proteins (Wisniewski et al., 1999). Antifreeze proteins (AFPs, also named ice-binding proteins or thermal hysteresis proteins) are able to bind to and prevent the growth of ice crystals (Jia and Davies, 2002). Found in a diversity of organisms, including plants, AFPs function by depressing the freezing point of ice crystals, or possibly by binding to heterogeneous ice nucleators to stabilize the supercooled state in an organism (Wilson and Leader, 1995). AFP activity can be measured in several ways, such as with a nanoliter osmometer (Ramlov, 2011), or by ice-recrystallization inhibition (Knight et al., 1995). A nanoliter osmometer consists of a cold stage on a microscope through which the growth of an ice crystal is observed. In the presence of an AFP, ice growth is arrested at subzero temperatures, and there is a separation between the melting point and the point at which the ice crystal begins to grow again. This temperature difference is termed thermal hysteresis, and is often used as an indicator of AFP activity. Ice recrystallization inhibition (IRI) relies on the observation that in the absence of AFP activity, small ice crystals shrink as larger ice crystals will grow. This technique is sensitive and can easily detect activity down in the sub-micromolar concentration (Knight et al., 1995).

Research on the peach dehydrin PCA60 has suggested that dehydrins may have AFP activity (Wisniewski et al., 1999). A protein extract from peach bark was shown to have weak thermal hysteretic activity. Attempts in our laboratory to demonstrate AFP activity in dehydrins have not been successful, suggesting that dehydrins do not have such activity. Several dehydrins were tested, including artificial K-concatemers, *V. riparia* K_2_ and YSK_2_, Dhn5 and even PCA60. No ability to inhibit ice crystal growth using ice-recrystallization inhibition (Hughes et al., 2013) or the nanoliter osmometer (unpublished results) was observed. These results suggest that the PCA60 activity is likely due to trace AFP contaminants, despite the effort made to purify the protein (isoelectric focusing and preparative acrylamide electrophoresis). Using bacterial recombinant dehydrins is an unlikely reason for the lack of activity in our laboratory since PCA60 does not contain an S-segment and therefore would not undergo any post-translational modification that could induce ice-binding activity. The lack of activity also fits with what is known about AFPs, which is that antifreeze proteins are highly rigid structures, often with a large number of hydrogen bond interactions to stabilize their structures at low temperatures (Graether and Sykes, 2004).

Many studies have examined the interaction between dehydrins and metal ions, including that by Svensson et al. (2000). They observed that the *Arabidopsis* dehydrins RAB18, LTI29, LTI30, and COR47 contain several histidine residues, which they exploited by purifying these dehydrins using immobilized metal affinity columns (IMAC; Svensson et al., 2000). They also tested several of the commonly used metal ions in IMAC (Ni(II), Cu(II), Co(II) and Zn(II)], and found that the proteins bind each metal with more or less the same affinity. One difference they observed is that LTI30 bound the strongest to the columns, which they attributed to the 11 His–His residues, a dipeptide that binds metals very strongly (Porath, 1992).

Metal binding was further used by other research groups to purify dehydrins (Ueda et al., 2003; Hara et al., 2005). Hara et al. (2005) performed an analysis of metal binding to measure the affinity of these ions for the citrus dehydrin CuCOR15. Fe(III), Co(II), Ni(II), Cu(II) and Zn(II) all bound to the dehydrin from citrus, while Mg(II), Ca(II) and Mn(II) did not, demonstrating that metal binding by dehydrins is not simply a non-specific charged interaction in which any cation can participate. The majority of the metals bound with a dissociation constant (K_d_) of ~1–2 μM, while Fe(III) bound nearly a thousand times more weakly (1.4 mM). Using synthetic peptides, Hara et al. (2005) showed that there is no ion binding to the K-segments, and that the strongest binding is to the sequence HKGEHHSGDHH, likely due to the His-His dipeptides. A similar study was carried out by Rahman et al. (2011) with TsDHN1 and TsDHN2 using isothermal calorimetry (ITC) to characterize metal binding. In the presence of Zn(II), it was shown that there were two zinc binding sites on TsDHN1 with a K_d_ of 45 μM, while there was one zinc binding site on TsDHN2 with a K_d_ of 26 μM. These affinities are slightly weaker than those observed above but are of a similar order of magnitude, which may be due to fewer His and His–His sequences in these proteins. In addition to His residues, phosphorylation of ERD14 has been shown to result in Ca(II) binding, most likely at the S-segment (Alsheikh et al., 2003). The authors speculate that this may allow some dehydrins to act as calcium buffers.

Dehydrins have been observed to undergo changes in structure and possibly oligomeric state when bound to metals. This effect appears to be dependent on the particular dehydrin examined. Work on the *A. thaliana* AtHIRD11 (Hara et al., 2013) and *T. salsuginea* TsDHN1 and TsDHN2 showed such changes. The study on AtHIRD11 used Cu(II), which caused self-association of the dehydrin (Hara et al., 2013). CD experiments showed a loss of signal at 200 nm, which the authors suggest represents the loss of disorder. The TsDHN1 and TsDHN2 studies also used CD and FTIR to examine for any structural change in the presence of Zn(II) (Rahman et al., 2010). However, an inspection of the CD plots suggests an alternate explanation. In both studies the authors report a propensity for the protein to aggregate and the CD spectra show a loss of coil signal but without a concomitant gain of other secondary structure such as α-helicity or β-strands. Therefore, one alternate explanation is that the loss of coil signal may be the loss of protein due to aggregation. One study showed no change in the structure of the *A*. *thaliana* Cor47, LTI29, and LTI30 until a very high concentration of metal (10 mM) was used, despite the proteins being fairly rich in His residues, including a number of His-His dipeptides (Mouillon et al., 2008). Once again, it may be that different dehydrins perform different protective roles in the plant.

One proposed role for metal binding by dehydrins has been to protect the plants from reactive oxygen species (Hara et al., 2005). Dehydrins would scavenge for the metals, since transition metals are involved in generating hydroxyl radicals. Hara et al. proposed that the increased metal concentration in the cytoplasm during dehydration could generate more radicals. The ability of dehydrins to protect from this has been tested both *in vitro* using a Cu-ascorbate system (Hara et al., 2013) and *in vivo* using *Brassica juncea* dehydrins BjDHN2 and BjDHN3 expressed in transgenic tobacco plants (Xu et al., 2008). Hara examined a panel of 27 dehydrin peptides from 14 different species to determine the relationship between the sequence and effectiveness in protection from the generation of reactive oxygen species. They showed that reducing the production of reactive oxygen species was dependent on the number of His residues present and the length of the peptide. The *in planta* study by Xu et al. (2008) transgenically expressed BjDHN2 and BjDHN3 (SK_2_ dehydrins) in tobacco plants. Exposure to heavy metals in plants had previously been shown to cause overexpression of SK_n_ dehydrins (Zhang et al., 2006; Xu et al., 2008). An ability to protect against heavy metal was suggested by the lowered electrolyte leakage and reduction in lipid peroxidation.

A wide variety of dehydrins have been found to be localized to the nucleus (see Table 2), which poses the question of whether dehydrins might bind to DNA. It was first proposed that the Y-segment might be involved in DNA binding since it has some sequence similarity with the ATP binding domains of the chaperone proteins GroEL and GroES (Close, 1996). The highest sequence similarity is to that of *E. coli* GroES (NDGYGVK) (Martin et al., 1993), however this theory of ATP binding by the Y-segment has yet to be supported by experimental evidence. Complicating this proposal is that several of the dehydrins that have been found in the nucleus do not have Y-segments. This could indicate that dehydrins play several roles in the nucleus, or that the Y-segment is not involved in DNA binding. When considering the similarity of the Y-segment to nucleotide-binding chaperones, it is important to note the differences between nucleotide binding and DNA binding. Not only are individual nucleotides much smaller and have several more surfaces available for interactions, but also ATP is an important energy carrier for the cell and therefore has uses that are unrelated to its role as a building block for nucleic acids.

Another DNA binding mechanism that has been investigated is the use of ions to mediate the interaction between dehydrins and nucleic acids. It was discovered that the citrus dehydrin CuCOR15 bound DNA and RNA in a non-specific manner in the presence of physiological concentrations of Zn(II) (Hara et al., 2009). Interestingly, binding was not significantly stimulated by other divalent cations. Following this discovery, the authors divided the CuCOR15 sequence into five different parts to narrow down the region(s) responsible for the interaction. When each domain was tested with a filter binding assay, it was determined that three domains contributed to the binding, all in a zinc-dependent manner (Hara et al., 2009). Two of the domains were rich in His, which is known to interact with metal ions through its imidazole ring. The other, even stronger-binding domain contained a Lys-rich segment. This sequence is different from the K-segment, which did not contribute significantly to binding to DNA. The authors suggested that the CuCOR15 dehydrin could protect nucleic acids from desiccation damage by coating and stabilizing DNA and RNA, and that this could explain why many dehydrins are located in both the cytoplasm and the nucleus: RNA is present in the cytoplasm, whereas DNA is located in the nucleus.

### Enzyme cryoprotection

An *in vitro* activity of dehydrins that could be indicative of how they protect plants from freezing stress *in vivo* is the cryoprotection of lactate dehydrogenase (LDH) (Carpenter and Crowe, 1988; Lin and Thomashow, 1992; Kazuoka and Oeda, 1994; Houde et al., 1995; Hara et al., 2001; Momma et al., 2003; Goyal et al., 2005; Reyes et al., 2008; Hughes and Graether, 2011; Drira et al., 2013). Presumably the same mechanism used to protect the model enzyme could protect plant enzymes from freeze/thaw damage during cold stress. In addition to LDH, α-amylase, an enzyme involved in starch degradation, has also been shown to be protected by dehydrins from cold damage (Rinne et al., 1999). When LDH is repeatedly frozen and thawed, it loses its activity due to denaturation and aggregation (Hughes and Graether, 2011). The addition of dehydrins or other cryoprotective proteins results in the recovery of enzyme activity. The results of the assay are often plotted as percent recovery of LDH activity vs. the logarithm of the protein concentration. The line shape of the plot is sigmoidal, showing that at low concentrations of protectant there is little or an undetectable amount of activity recovery, while at high concentrations usually all of the LDH activity is recovered.

In our examination of the literature in the field, we found it challenging to compare the relative effectiveness of different dehydrins. Our goal was to determine which YSK architecture was the most efficient at protecting LDH from being damaged. Efficiency is typically expressed as PD_50_ value, which represents the concentration of additive required to recover 50% of LDH activity. A lower value would therefore represent a more efficient protection of activity. We observed that the PD_50_ values of BSA varied between research groups by as much as ten-fold [compare the data in Houde et al. (1995) with Wisniewski et al. (1999)]. This is likely a reflection of different freeze/thaw protocols, such as the freezing method, thawing method, choice of buffer, and choice of LDH source.

Because of these complications, we compared a large number of different dehydrins of different sizes in one LDH cryoprotection assay system to determine what role the K-segments or the size of the dehydrin may have on the PD_50_ value. We firstly used artificial constructs of K and KK peptides (i.e., K_2_ without the ϕ-segment) to compare to *V. riparia* K_2_ in order to directly determine the importance of the K-segments in this assay. The rationale being that if the K-segments are relevant, the KK peptide would have approximately the same activity as K_2_, since they both have two K-segments, whereas the K-peptide should have approximately half of the activity of K_2_. The results showed that the K_2_ was more efficient at recovery than the KK-peptide, and considerably more efficient that the K-peptide, demonstrating that all of the protein contributes to the protection.

It has been suggested that the K-segments are important in the cryoprotection assay (Reyes et al., 2008; Drira et al., 2013). However, this may be due to a loss of size of the protein rather than the K-segment alone (Hughes et al., 2013). Our proposal is that dehydrins act as molecular shields, in which case the protein prevents partially denatured LDH molecules from coming together and aggregating. Using artificial concatemers and natural dehydrins covering a large molecular weight range, it was shown that the there is a correlation between the hydrodynamic (Stoke's) radius of the protein and its efficiency. The superior ability of dehydrins to protect LDH from freeze/thaw damage suggests that disorder may play a role in cryoprotection. To test this, polyethylene glycol (PEG) molecules of similar hydrodynamic radii was used in the same assay (Hughes et al., 2013). The results showed the same level of protection as the dehydrins, demonstrating that disorder is important for effective cryoprotection of LDH. The mechanism by which dehydrins protect LDH from damage has also been examined (Hughes and Graether, 2011). Using NMR, it was shown that the dehydrin does not bind to the enzyme. These experiments provide proof that dehydrins function in the cryoprotection assays by acting as molecular shields.

## Conclusion

This review has focused on *in vitro* dehydrin studies in order to investigate the protein's potential roles (membrane interaction, enzyme cryoprotection, reactive oxygen species scavenging, and interaction with DNA) in the cell. The next step is to take these *in vitro* functions and test them in more complex *ex vivo* and *in vivo* experimental systems in order to determine which proposed roles are true representations of the natural function of dehydrins. It has been shown several times that the transgenic expression of dehydrins in plants can lead to increased cold and drought tolerance (Hara et al., 2003; Puhakainen et al., 2004; Shekhawat et al., 2011; Xie et al., 2012; Yang et al., 2014), but the gap between *in vitro* experiments and *in vivo* function has yet to be bridged.

### Conflict of interest statement

The authors declare that the research was conducted in the absence of any commercial or financial relationships that could be construed as a potential conflict of interest.
